# Peritoneal Air Exposure Elicits an Intestinal Inflammation Resulting in Postoperative Ileus

**DOI:** 10.1155/2014/924296

**Published:** 2014-07-22

**Authors:** Shanjun Tan, Wenkui Yu, Zhiliang Lin, Qiyi Chen, Jialiang Shi, Yi Dong, Kaipeng Duan, Xiaowu Bai, Lin Xu, Jieshou Li, Ning Li

**Affiliations:** ^1^Research Institute of General Surgery, Jinling Hospital, Medical School of Nanjing University, 305 East Zhongshan Road, Nanjing 210002, China; ^2^Research Institute of General Surgery, Jinling Hospital, Clinical School of Nanjing, Second Military Medical University, 305 East Zhongshan Road, Nanjing 210002, China

## Abstract

*Background.* The pathogenesis of postoperative ileus (POI) is complex. The present study was designed to investigate the effects of peritoneal air exposure on the POI intestinal inflammation and the underlying mechanism.* Methods.* Sprague-Dawley rats were randomized into five groups (6/group): the control group, the sham group, and three exposure groups with peritoneal air exposure for 1, 2, or 3 h. At 24 h after surgery, we analyzed the gastrointestinal transit, the serum levels of tumor necrosis factor (TNF)-*α*, interleukin (IL)-1*β*, IL-6, and IL-10, the myeloperoxidase activity, and the levels of TNF-*α*, IL-1*β*, IL-6, and IL-10 in the ileum and colon. The oxidant and antioxidant levels in the ileum and colon were analyzed by measuring malondialdehyde (MDA), superoxide dismutase (SOD), glutathione peroxidase (GSH-Px), and total antioxidant capacity (T-AOC).* Results.* Peritoneal air exposure caused an air-exposure-time-dependent decrease in the gastrointestinal transit. The length of peritoneal air exposure is correlated with the severity of both systemic and intestinal inflammations and the increases in the levels of MDA, SOD, GSH-Px, and T-AOC.* Conclusions.* The length of peritoneal air exposure is proportional to the degree of intestinal paralysis and the severity of intestinal inflammation, which is linked to the oxidative stress response.

## 1. Introduction

Postoperative ileus (POI) is a common and major iatrogenic complication after surgery, especially open abdominal surgery [1]. Patients with POI often have unique clinical features, including abdominal distension and bloating, with a mix of nausea and vomiting and delayed passage of flatus and stool [2]. Due to a delayed recovery to normal intestinal motility, patients often have prolonged hospital stay, increasing medical costs and affecting postoperative rehabilitation [3, 4]. Unfortunately, the etiology of POI has not been fully elucidated [1]. There are many factors affecting the development and prognosis of POI, including the operation conditions and procedures and the severity of inflammation [1, 5]. The intestinal inflammation plays an important role in the development and progression of POI [6–8], showing a significant correlation between the postoperative increase in the intestinal inflammatory response and the decreased gastrointestinal motility [9, 10].

Peritoneal air exposure is a common clinical phenomenon in abdominal surgery, impacting the patients' well-being after open abdominal operation. However, this problem has long been ignored. Several studies have shown that open abdominal surgery is associated with postoperative systemic inflammatory response, leading to POI, and that laparoscopic surgery results in significantly less systemic inflammatory response after surgery and a much shorter period of POI [11–14]. Although the difference in the degree of surgical trauma has been directly linked to the different outcomes between open and laparoscopic surgery, the exposure of peritoneal cavity to air may be a key factor as well [15].

There is a long-standing interest in exploring the effects of laparotomy on the body's responses, including inflammatory response and organ functions. Several studies have demonstrated that exposure of the peritoneal cavity to air can induce the peritoneal and systemic inflammatory responses to laparotomy without any procedure [16–19]. Patients often need a long time to return to passage of flatus and stool, although they just undergo a minor open abdominal operation. These findings suggest that peritoneal air exposure may induce intestinal inflammation and play an important role in the development and progression of POI. However, little is known about the effect of peritoneal air exposure on intestinal inflammation and its further contribution to POI. Additionally, an early study indicates that the pleural oxygen exposure in thoracotomy results in oxidative stress response leading to the lung and systemic inflammations [20]. It is unknown if the oxidative stress response is involved in the intestinal inflammation after air exposure in open abdominal surgery.

The present study was designed to investigate whether the duration of peritoneal air exposure affects the POI intestinal inflammation and to determine the underlying mechanisms, including the role of oxidative stress responses. In the present study, we emphasized the relationship between peritoneal air exposure during operation and POI, which has not been directly determined in previous studies. The investigation on the relationship between duration of peritoneal air exposure and the inflammatory responses as well as POI has a direct clinical implication, especially under the experimental conditions mimicking the clinical operation environment. In addition, we investigated the mechanisms responsible for the prolonged peritoneal air exposure-induced inflammatory responses, including the response to oxidative stress.

## 2. Materials and Methods

### 2.1. Animals

Male Sprague-Dawley rats (weighing 210–230 g) were obtained from the Animal Research Center, Jinling Hospital, Nanjing, China. The rats were housed in a temperature-controlled room on a 12 h/12 h light/dark cycle, with free access to standard rat chow and tap water* ad libitum*. All the animals were allowed a minimum of one-week acclimatization before study. This animal protocol was reviewed and approved by the Institutional Animal Care and Use Committee of Jinling Hospital. All the experiments were performed according to the National Institutes of Health Guidelines on the use of laboratory animals.

### 2.2. Experimental Groups and Surgical Procedures

Thirty rats were randomly divided into five groups (6/group): the control group, the sham group, and three exposure groups with peritoneal air exposure for 1, 2, or 3 h after operation, respectively. The animals were anesthetized by the subcutaneous injection of 2% pentobarbital sodium (3.5 mL/kg) [21]. The surgical procedures were performed in an aseptic and temperature- and humidity-controlled environment, in order to avoid possible confounding factors such as foreign pathogens, dehydration, and hypothermia. For the three exposure groups, after the anesthesia was induced, a 3 cm midline abdominal incision was made [18], and the wound edge was then retracted to allow for maximal peritoneal air exposure for 1, 2, and 3 h, respectively [17]. To reduce possible dehydration, an intraoperative peritoneal spraying of physiological saline (5 mL) was performed in a 30 min interval. At the end of air exposure, the abdomen was closed in one layer with 3–0 silk continuous suture. For the sham group, the animals underwent the same anesthesia and operation procedure, but without laparotomy. For the control group, the animals underwent the same anesthesia, but without operative procedures. The animals in all groups were monitored until recovery from anesthesia and then returned to their assigned cages.

### 2.3. Determination of Gastrointestinal Transit and Intestinal Tissue Sampling

At 24 h after surgery, the gastrointestinal transit was determined by the charcoal transport assay as previously described by Li et al. [8]. Briefly, the rats were administered with a black marker (the 10% charcoal suspension in 10% gum Arabic, 10 mL/kg) by gavage. 20 min later, the rats were anesthetized by the subcutaneous injection of 2% pentobarbital sodium and the blood samples were collected immediately from the inferior vena cava for cytokine analyses; and then, the entire small intestine from the pylorus to the cecum was removed and measured. The distance travelled by the marker in the small intestine was measured and reported as a percentage of length of the entire small intestine. Subsequently, representative segments of terminal ileum and proximal colon were harvested individually for analyses of cytokines, gene expression, myeloperoxidase (MPO) activity, and oxidant and antioxidant levels.

### 2.4. Determination of Concentrations of Cytokines in Serum and Tissues of Ileum and Colon

The serum was prepared by centrifugation at 1,500 rpm for 15 min at 4°C. The tissues of ileum and colon were separately homogenized and centrifuged at 4,000 rpm for 15 min at 4°C, and then, the supernatants were transferred to a clean tube before analysis. The concentrations of cytokines tumor necrosis factor (TNF)-*α*, interleukin (IL)-1*β*, IL-6, and IL-10 in the serum and the supernatants of tissues of ileum and colon were determined using an ELISA kit for rats (R&D Systems, Germany) according to the manufacturer's instructions. The values were expressed as pg/mL in the serum or pg/g protein in the tissues.

### 2.5. Analysis of Cytokine Gene Expression in Tissues of Ileum and Colon

The total RNA was isolated using Trizol reagent (Invitrogen, USA) according to the manufacturer's instructions. The mRNAs of TNF-*α*, IL-1*β*, IL-6, and IL-10 were separately reverse-transcribed to cDNA and measured using real-time polymerase chain reaction (RT-PCR) as described in our previous studies [22–24]. Glyceraldehyde-3-phosphate dehydrogenase (GAPDH) was used as an internal standard to normalize the target mRNAs, and relative quantifications and calculations were performed by the 2^−ΔΔCT^ method to analyze gene expression levels [25]. The primer sequences are listed in Table 1.

### 2.6. Assessment of MPO Activity in Tissues of Ileum and Colon

The tissues of ileum and colon were separately homogenized and centrifuged at 4,000 rpm for 15 min at 4°C, and then the supernatants were obtained. The MPO activity was quantitatively measured by spectrophotometry at 460 nm as described in our previous study [24]. The values were expressed as units/g protein in the tissues.

### 2.7. Analyses of Malondialdehyde (MDA), Superoxide Dismutase (SOD), Glutathione Peroxidase (GSH-Px), and Total Antioxidant Capacity (T-AOC) in Tissues of Ileum and Colon

The levels of MDA, SOD, GSH-Px, and T-AOC in the supernatants of tissues homogenates were determined with the commercial analysis kits (Nanjing Jiancheng Biocompany, China), according to the manufacturer's instructions. The MDA levels were expressed as nmol/mg protein in the tissues. The levels of SOD, GSH-Px, and T-AOC were expressed as units/mg protein in the tissues.

### 2.8. Statistical Analysis

The experimental data were expressed as mean ± SD. Statistical analyses were performed using SPSS 17.0 software (SPSS Inc., USA). The comparisons of differences among the various groups were accomplished by using one-way analysis of variance (ANOVA) after homogeneity test for variance. Significant results were further analyzed post hoc using the Dunnett test. Differences were considered statistically significant when *P* < 0.05.

## 3. Results

### 3.1. General Observations

All rats survived the entire experiments. No significant differences were found in body weight, behavior, and postoperative fluid intake among various groups.

### 3.2. Gastrointestinal Transit

**Figure 1:**
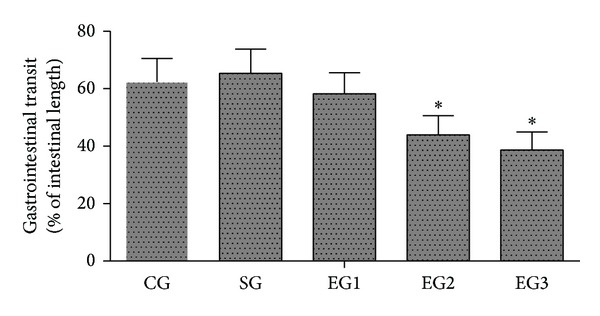
Gastrointestinal transit. CG: control group; SG: sham group; EG1, EG2, and EG3: exposure groups with peritoneal air exposure for 1, 2, and 3 h, respectively. The data are expressed as mean ± SD, *n* = 8. *P < 0.05, versus the CG group.

As shown in Figure 1, there were no significant differences in gastrointestinal transit between the control and sham groups (*P* > 0.05). Peritoneal air exposure induced a progressive decrease in gastrointestinal transit; the differences were significant in the 2 h and 3 h exposure groups when compared with the control group (*P* < 0.05).

### 3.3. Levels of Cytokines TNF-*α*, IL-1*β*, IL-6, and IL-10 in Serum

**Figure 2:**
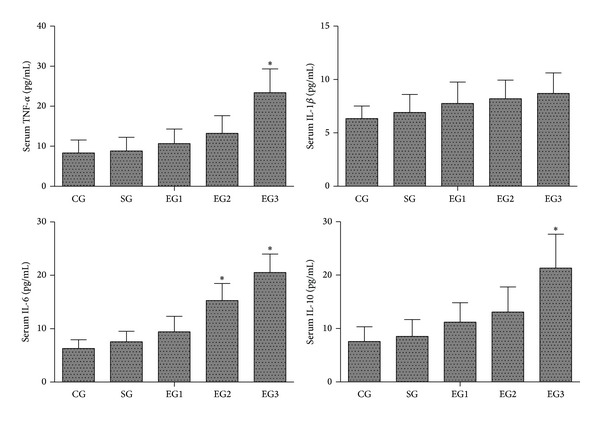
Serum concentrations of inflammatory cytokines TNF-*α*, IL-1*β*, IL-6, and IL-10 in each group. CG: control group; SG: sham group; EG1, EG2, and EG3: exposure groups with peritoneal air exposure for 1, 2, and 3 h, respectively. Data are expressed as mean ± SD, *n* = 8. *P < 0.05, versus the CG group.

As shown in Figure 2, there were no significant differences in the serum concentrations of TNF-*α*, IL-1*β*, IL-6, and IL-10 between the control and sham groups. Peritoneal air exposure elicited a progressive increase in the serum concentrations of TNF-*α*, IL-1*β*, and IL-6. In the 1 h exposure group, the serum concentrations of these proinflammatory cytokines increased slightly, but did not reach the statistical significance level. In the 2 h exposure group, the serum concentrations of IL-6 were significantly higher than those of the control group (*P* < 0.05). In the 3 h exposure group, the serum TNF-*α* and IL-6 concentrations were significantly increased, compared with those of the control group (*P* < 0.05), but no significant differences were found in the concentrations of IL-1*β*. In addition, the anti-inflammatory cytokine IL-10 levels were only increased in the 3 h exposure group, compared with those of the control group (*P* < 0.05).

### 3.4. Concentrations of TNF-*α*, IL-1*β*, IL-6, and IL-10 in Tissues of Ileum and Colon

**Figure 3:**
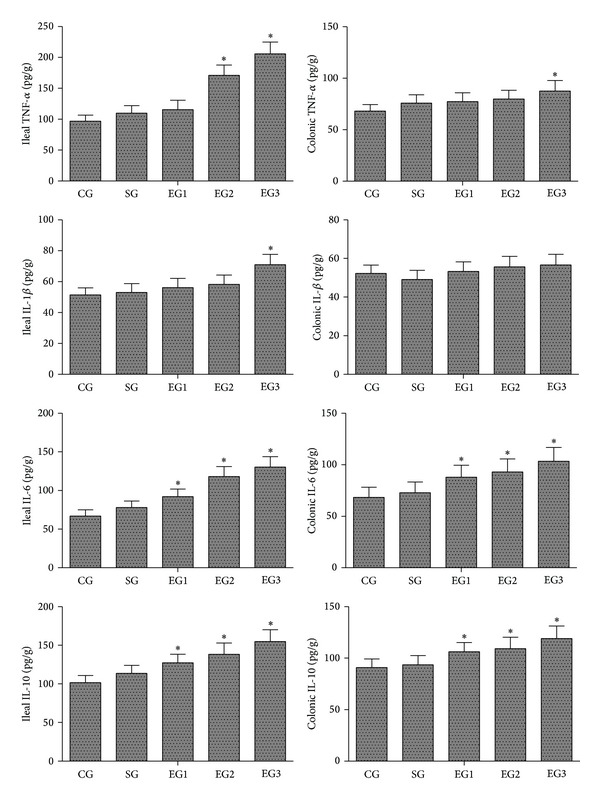
Concentrations of TNF-*α*, IL-1*β*, IL-6, and IL-10 in the tissues of ileum and colon. CG: control group; SG: sham group; EG1, EG2, and EG3: exposure groups with peritoneal air exposure for 1, 2, and 3 h, respectively. Data are expressed as mean ± SD, *n* = 8. *P < 0.05, versus the CG group.

As shown in Figure 3, there were no significant differences in the concentrations of TNF-*α*, IL-1*β*, IL-6, and IL-10 in the tissues of ileum and colon between the control and sham groups. Peritoneal air exposure elicited a progressive increase in the tissue concentrations of TNF-*α*, IL-1*β*, and IL-6. The levels of IL-6 in the tissue of both ileum and colon were significantly increased in all exposure groups, compared with those of the control group (*P* < 0.05). The TNF-*α* levels in the ileum in the 2 and 3 h exposure groups and in the colon in the 3 h exposure group were significantly higher than those of the control group (*P* < 0.05). Meanwhile, the ileal concentrations of IL-1*β* were significantly increased in the 3 h exposure group, compared with those of the control group (*P* < 0.05), although there were no significant differences in colonic concentrations of IL-1*β* among all the groups. In addition, peritoneal air exposure also elicited a significant increase in both ileal and colonic concentrations of anti-inflammatory cytokine IL-10 in all exposure groups, compared with those of the control group (*P* < 0.05).

### 3.5. Gene Expression of TNF-*α*, IL-1*β*, IL-6, and IL-10 in Tissues of Ileum and Colon

**Figure 4:**
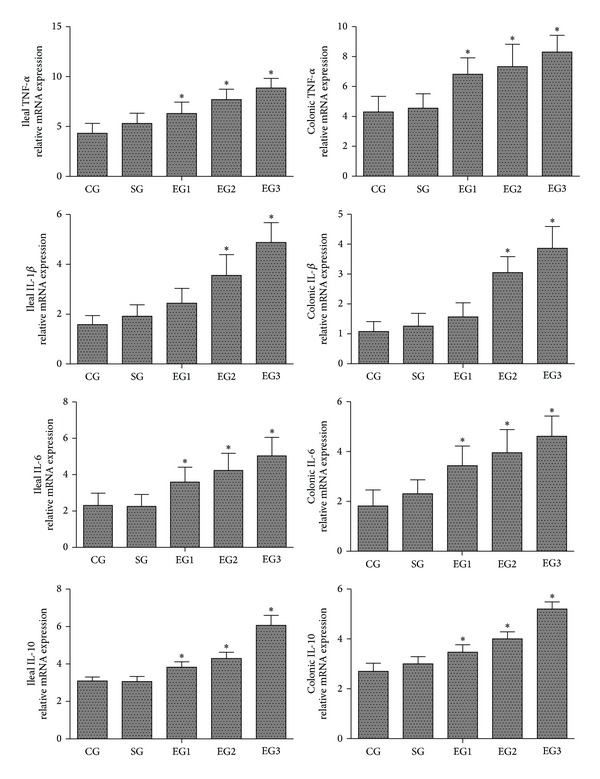
Relative mRNA expression of TNF-*α*, IL-1*β*, IL-6, and IL-10 in the tissues of ileum and colon. CG: control group; SG: sham group; EG1, EG2, and EG3: exposure groups with peritoneal air exposure for 1, 2, and 3 h, respectively. Data are expressed as mean ± SD, *n* = 8. *P < 0.05, versus the CG group.

As shown in Figure 4, there were no significant differences in the gene expression of TNF-*α*, IL-1*β*, IL-6, and IL-10 in the tissues of ileum and colon between the control and sham groups. Peritoneal air exposure induced a significant upregulation of gene expression of TNF-*α*, IL-1*β*, IL-6, and IL-10 in the tissues of ileum and colon in all exposure groups except IL-1*β* in the 1 h exposure group when compared with that of the control group (*P* < 0.05).

### 3.6. MPO Activity in Tissues of Ileum and Colon

**Figure 5:**
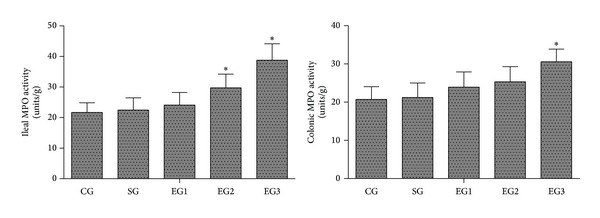
MPO activity in the tissues of ileum and colon. CG: control group; SG: sham group; EG1, EG2, and EG3: exposure groups with peritoneal air exposure for 1, 2, and 3 h, respectively. Data are expressed as mean ± SD, *n* = 8. *P < 0.05, versus the CG group.

As shown in Figure 5, there were no significant differences in the MPO activity in the tissues of ileum and colon between the control and sham groups. Peritoneal air exposure induced a progressive increase in the MPO activity in the tissues of ileum and colon. The ileal MPO activity in the 2 and 3 h exposure groups and the colonic MPO activity in the 3 h exposure group were significantly higher than those of the control group (*P* < 0.05).

### 3.7. The Levels of MDA, SOD, GSH-Px, and T-AOC in Tissues of Ileum and Colon

**Figure 6:**
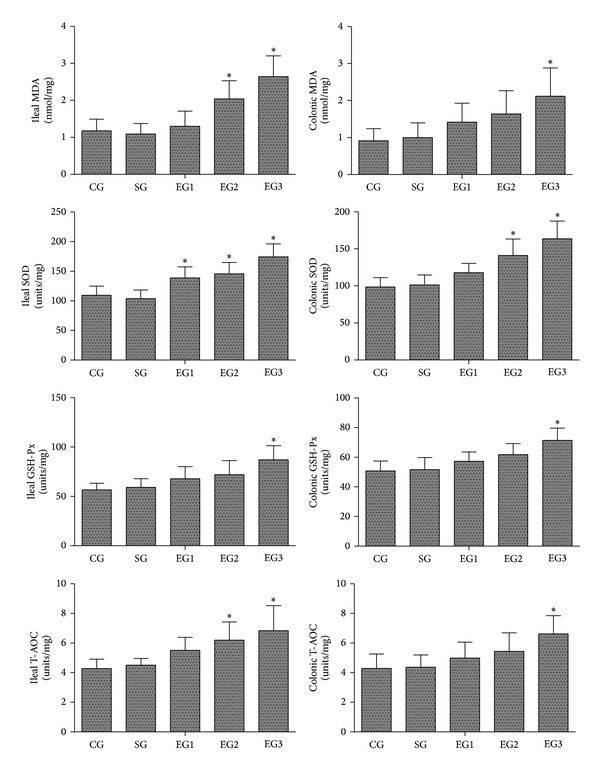
The levels of MDA, SOD, GSH-Px, and T-AOC in the tissues of ileum and colon. CG: control group; SG: sham group; EG1, EG2, and EG3: exposure groups with peritoneal air exposure for 1, 2, and 3 h, respectively. Data are expressed as mean ± SD, *n* = 8. *P < 0.05, versus the CG group.

As shown in Figure 6, there were no significant differences in the levels of MDA, SOD, GSH-Px, and T-AOC in the tissues of ileum and colon between the control and sham groups. Peritoneal air exposure elicited a progressive increase in the tissue levels of MDA, SOD, GSH-Px, and T-AOC. The ileal MDA activity in the 2 and 3 h exposure groups and the colonic MDA activity in the 3 h exposure group were significantly higher than those of the control group (*P* < 0.05). The ileal SOD activity in all the three exposure groups and the colonic SOD activity in the 2 and 3 h exposure groups were significantly higher than those of the control group (*P* < 0.05). For the GSH-Px levels, there were no significant differences between the 1 and 2 h exposure groups and the control group, but the ileal and colonic GSH-Px levels in the 3 h exposure group were significantly higher than those of the control group (*P* < 0.05). Additionally, the ileal T-AOC levels in the 2 and 3 h exposure groups and the colonic T-AOC level in the 3 h exposure group were significantly higher than those of corresponding control group (*P* < 0.05).

## 4. Discussion

In the present study, we investigated the relationships between the duration of peritoneal air exposure and changes in the intestinal inflammation related cytokines, the gastrointestinal motility, and the oxidative stress responses in rats. The results demonstrated that the increases in the length of peritoneal air exposure caused a progressive decrease in gastrointestinal transit and progressive increases in the systemic and intestinal inflammation and oxidative stress responses.

POI is a common clinical problem after almost every abdominal surgery, often leading to increased patient morbidity and delayed rehabilitation [5]. The majority of the published reports suggest that POI is a transient gastrointestinal motility disorder involved in response to surgical stress [1, 5, 8, 26]. Enhancement of gastrointestinal motility will significantly improve POI [27, 28]. To elucidate the effect of peritoneal air exposure on POI, we assessed the gastrointestinal motility, using the charcoal transport assay, a commonly used objective measurement for the gastrointestinal motility in animal studies [29]. It has been demonstrated that POI is associated with a decreased gastrointestinal transit, as assessed by charcoal transport [8]. In the present study, we found that peritoneal air exposure induced a significant decrease in the gastrointestinal transit in all exposure groups, reaching statistical significance level in the 2 and 3 h exposure groups, indicating that peritoneal air exposure is an important cause for POI.

It is well accepted that intestinal inflammation caused by surgical stress plays an important role in the development and progression of POI [6–8]. Proinflammatory cytokines including TNF-*α*, IL-1*β*, and IL-6 are rapidly induced in POI animals [6, 30, 31]. They act as depressant factors and contribute to the decreased gastrointestinal motility through various mechanisms such as direct cytotoxic effects and induction of nitric oxide (NO) and prostanoids [26]. In contrast, anti-inflammatory cytokine IL-10 downregulates inflammatory processes to avoid an excessive and prolonged inflammation [26]. Stoffels et al. [32] report that IL-10 deficiency results in a prolonged postoperative intestinal dysmotility and abnormal high gene expression of proinflammatory mediators in POI induced by intestinal surgical manipulation in mice. MPO is an enzyme abundantly stored in azurophilic granules of neutrophils and is released into extracellular fluid in the inflammatory process. Therefore, MPO activity is a prognostic biomarker for inflammatory response in a variety of acute and chronic inflammatory conditions [33].

There are many factors affecting the development and progression of POI after intestinal trauma, including effects of dehydration and hypothermia on intestinal inflammation. In the present study, we set up our experimental conditions mimicking the clinical operation environment such as well-controlled room temperature and humidity. In order to reduce possible dehydration, we performed intraoperative peritoneal spraying of physiological saline (5 mL each) in a 30 min interval. We also monitored the body temperature of the animals to demonstrate there was no hypothermia in these animals. Therefore, we believe that, after controlling the major factors that may affect the inflammatory response, the increased POI inflammation and oxidative stress response and the decreased gastrointestinal motility observed in the exposure groups could be attributed to the air exposure in a time-dependent manner.

In the present study, we demonstrated that the serum and intestinal protein concentrations of proinflammatory cytokines TNF-*α*, IL-1*β*, and IL-6 were significantly increased in proportion to the time length of peritoneal air exposure. Similar findings were obtained with the analyses of the MPO activity and the gene expression of TNF-*α*, IL-1*β*, and IL-6 in ileum and colon. Surprisingly, the level of anti-inflammatory cytokine IL-10 was also progressively increased with time length of peritoneal air exposure. The underlying mechanism for the IL-10 increase was not investigated in the present study; we speculate that there may be a compensation or feedback mechanism for the IL-10 increase in response to prolonged and intensive increases in proinflammatory cytokines in the exposure animals. In summary, we believe that peritoneal air exposure could elicit a general activation of systemic and local intestinal inflammation, in an exposure time-dependent manner.

There is an interest in determining the time-dependent cytokine responses to intestinal trauma, which may be helpful in the development of biomarkers and intervention approaches to prevent and treat POI inflammation and facilitate patient's recovery after open abdominal surgery. For example, Wehner et al. examined the induction of IL-6 after intestinal surgical stress (20 min) in rats [31], demonstrating an early release of IL-6 after an intestinal trauma. They reported that the IL-6 mRNA levels peaked at 1 h after intestinal manipulation (IM), remained significantly increased at 3 h after surgical stress, and then regressed toward the level of sham operated animals and controls after 12 h and 24 h, respectively. In the present study, we observed the significantly elevated IL-6 levels at 24 h after a laparotomy without traumatizing the intestine. There were apparent differences in the duration of IL-6 elevation between the previously reported results [31] and our results from the present study. Although the exact reasons for the differences are not clear, we speculate the possible factors may include the following: (1) the operation procedure and the duration of intestinal trauma (20 min in previous study versus 1–3 h in the present study); (2) the degrees of possible dehydration (possibly greater in the present study); and (3) the intestinal tissues used in the analysis of the cytokines (muscularis externa (ME) in their study versus the entire intestine in the present study). In addition, our pilot study demonstrated that IL-6 was elevated at 3 h after peritoneal air exposure (data not shown). Therefore, in the present study, we only analyzed the 24 h samples, demonstrating a prolonged inflammatory response in the exposure animals.

The present study also demonstrated the increased oxidative responses in POI animals. The oxidative injury can occur during surgery when free radical oxygen species are increased to the level exceeding the body's normal elimination capacity, causing cellular oxidative stress response and injury [21]. There are several reports demonstrating that oxidative injury induces inflammatory response or increases the magnitude of the response, causing organ dysfunction [34–36]. Our results from the present study demonstrated that the levels of MDA, SOD, GSH-Px, and T-AOC in tissues of ileum and colon were increased after air exposure in rats. Increases in both oxidant and antioxidant levels indicate the elevated oxidative stress response in these animals. In addition, an early study indicates that the pleural oxygen exposure to normal air containing 21% oxygen in thoracotomy results in oxidative stress response leading to the lung and systemic inflammations [20]. We speculate that the oxidative stress response observed in the present study could be linked to air exposure in these experimental animals.

Previous studies have focused mainly on the surgical intestinal manipulation, inducing intestinal inflammation and, further, resulting in POI [6–8]. The extent of surgical intestinal manipulation causes the degree of intestinal inflammation and further determines the duration and severity of decreased gastrointestinal motility during POI [9]. These results indicate that surgical intestinal manipulation plays a key role in the induction of POI. However, in clinical practice, laparoscopic surgery has a shorter period of POI, compared with open abdominal surgery [11–14]. Further studies reveal that the advantage of this faster postoperative recovery of gastrointestinal motility may be due to the nonexposure of peritoneal cavity to air [15]. The present study directly showed that peritoneal air exposure could induce intestinal inflammation and further lead to a decrease in gastrointestinal transit. Therefore, when patients undergo excessive abdominal surgery, prevention of peritoneal air exposure may be an effective way to promote postoperative intestinal peristalsis and enhance recovery after surgery.

Of note, the present study had some limitations. For instance, the gastrointestinal transit is best quantified using a fluorescent marker quantified in 15 segments of the gastrointestinal tract to calculate a geometric center value [37]. In the present study, we used the leading edge analysis. Although this method has been used in many previous studies [8, 38–40], it has some limitations in sensitivity and specificity. We believe that the leading edge method met our purpose of the present study that was to compare the POI inflammation among different lengths of air exposure (1, 2 and 3 h), demonstrating that the decreased gastrointestinal motility was air-exposure-time-dependent. We speculate that, even if the method was not as accurate as the fluorescence method, the conclusion should be regarded as correct. Nevertheless, the fluorescence method should be used in future studies. In addition, it would be worth exploring the contribution of intestinal desiccation to the POI inflammatory response. In the present study, our goals were to explore the effects of the length of air exposure on the POI inflammatory responses and to determine possible mechanisms of action, including oxidative stress. The experimental conditions for the three exposure groups (1–3 h) were identical and kept consistent between the groups in order to avoid dehydration and hypothermia, but we did not directly determine the effects of dehydration and hypothermia on the POI inflammatory responses. Future studies in this field of research should include a group of animals with a 3 h laparotomy that are exposed to an arm mist of lactated Ringers, which would clearly show if intestinal desiccation-induced injury to the gut wall during the laparotomy/surgery is the major factor for POI inflammatory responses.

In the present study, we analyzed small bowel and colonic tissue for proinflammatory gene expression and influx of MPO positive cells. It has been suggested that inflammation leading to POI takes place mainly in the ME [6, 8–10, 37]. However, when we designed the current experiments, we were not sure if only ME was critical and other sections were not, since we had prolonged air exposure in the present study. Considering that there were many differences in experimental design, especially the duration of air exposure and the exposure conditions between our study and previous studies, we decided to use the entire intestine to analyze the cytokines and oxidative stress responses. It should be considered that, if we just analyzed the ME, the changes might be different from those of the entire intestine as observed in the present study. However, we speculate that the POI might be linked to the entire intestine under our experimental conditions, but we should test if the ME is the only and critical site in future studies.

As aforementioned, the present study focused on the etiology of POI; the possible intervention approach was not investigated. In the last decade there have been several publications dealing with the consequences of peritoneal gas exposure during laparotomy and/or laparoscopy, demonstrating that an enhanced local and systemic cytokine release can be seen after air exposure, which can be reversed by using a CO_2_ or CO enriched atmosphere [17, 41, 42]. Therefore, the question arises whether peritoneal CO or CO_2_ exposure is able to avoid intestinal inflammation and POI compared to air exposure. This should be clarified in future experiments.

In conclusion, our results suggest that the length of peritoneal air exposure is proportional to the degree of intestinal paralysis and the activation of intestinal inflammation, leading to POI. In addition, increased oxidative stress response is linked to peritoneal air exposure, which may lead to systemic and local intestinal inflammatory responses. Future studies should determine the exact molecular mechanism underlying the systemic and intestinal inflammation after peritoneal air exposure. Nevertheless, avoiding prolonged air exposure in open abdominal surgery should be encouraged in the clinical practice.

## Figures and Tables

**Table 1:** The primer sequences used for RT-PCR.

Target gene		PCR primer sequence	PCR product (bp)
GAPDH	Forward	5′-GGCATTGCTCTCAATGACAA-3′	223
	Reverse	5′-TGTGAGGGAGATGCTCAGTG-3′	
TNF-*α*	Forward	5′-ACTCCCAGAAAAGCAAGCAA-3′	211
	Reverse	5′-CGAGCAGGAATGAGAAGAGG-3′	
IL-1*β*	Forward	5′-AGGCTTCCTTGTGCAAGTGT-3′	230
	Reverse	5′-TGAGTGACACTGCCTTCCTG-3′	
IL-6	Forward	5′-CTGCTCTGGTCTTCTGGAGT-3′	229
	Reverse	5′-GGTCTTGGTCCTTAGCCACT-3′	
IL-10	Forward	5′-ATAACTGCACCCACTTCCCA-3′	231
	Reverse	5′-TTTCTGGGCCATGGTTCTCT-3′	
